# Frailty and anticoagulants in older subjects with atrial fibrillation: the EUROSAF study

**DOI:** 10.1093/ageing/afad216

**Published:** 2023-11-27

**Authors:** Alberto Pilotto, Nicola Veronese, Maria Cristina Polidori, Timo Strandberg, Eva Topinkova, Alfonso J Cruz-Jentoft, Carlo Custodero, Mario Barbagallo, Stefania Maggi, the EUROSAF Study Investigators, Alberto Ferri, Alessandra Argusti, Federica Gandolfo, Clarissa Musacchio, Katerin Leslie Quispe Guerrero, Alberto Pilotto, Carlo Custodero, Vincenzo Solfrizzi, Carlo Sabbà, Maria Cristina Polidori, Joshua Verleysdonk, Nico Noetzela, Timo Strandberg, Juhani Rossinen, Laura Pikkarainen, Tuomo Nieminen, Eva Topinkova, Helena Michalkova, Pavla Madlova, Lucie Bautzka, Stefania, Nicola Ferrara, Lucia Gioia, Anna Maria Iannicelli, Mario Barbagallo, Nicola Veronese, Giovanna Di Bella, Federica Cacioppo, Giovanni Ruotolo, Alberto Castagna, Regina Roller-Wirnsberger, Christian Sebesta, Sonja Lindner, Alfonso Cruz-Jentoft, Luisa A Hernandez-Sanchez, Jana Albeniz Lopez, Genesis Estefanıa Olaya-Loor, Pedro Marques da Silva, Heidi Gruner, Jean Petermans, Sophie Gillain, Veronique Jonart, Ondrej Vyska, Jiri Nakladal, Katarina Bielakova, Hana Matejovska-Kubesova, Adrian Enica, Stephanie Roth, Benjamin Jacquet, Vito Curiale, Nicolas Berg, Livia Mirea Cimpeanu, Rafaela Verissimo, Leonor Silva, Luciana Silva, Pedro Magalhães, Gabriel Ioan Prada, Anna Marie Herghelegiu, Catalina Raluca Nuta, Blanca Garmendia-Prieto, Isabel Lozano-Montoya, Javier Jaramillo-Hidalgo, Javier Gomez-Pav, Ursula Müller-Werdan, Gordon Werth, Adrian Rosada, Ozlem Yilmaz, Francesco Mattace-Raso, Sena Geurkaş

**Affiliations:** Geriatrics Unit, Department of Geriatric Care, OrthoGeriatrics and Rehabilitation, E.O. Galliera Hospital, Genova, Italy; Department of Interdisciplinary Medicine, University of Bari “Aldo Moro”, Bari, Italy; Geriatrics Unit, Department of Internal Medicine, University of Palermo, Palermo, Italy; Ageing Clinical Research, Department II of Internal Medicine and Center for Molecular Medicine Cologne, Faculty of Medicine and University Hospital Cologne, University of Cologne, Cologne, Germany; University of Helsinki and Helsinki University Hospital, Helsinki Finland; Center for Life Course Health Research, University of Oulu, Oulu, Finland; First Faculty of Medicine, Charles University in Prague, Czech Republic; Servicio de Geriatría, Hospital Universitario Ramón y Cajal (IRYCIS), Madrid, Spain; Department of Interdisciplinary Medicine, University of Bari “Aldo Moro”, Bari, Italy; Geriatrics Unit, Department of Internal Medicine, University of Palermo, Palermo, Italy; National Research Council, Neuroscience Section, Padova, Italy

**Keywords:** atrial fibrillation, frailty, multidimensional prognostic index, mortality, stroke, older people

## Abstract

**Aims:**

Literature regarding anticoagulants in older people affected by atrial fibrillation (AF) is limited to retrospective studies, poorly considering the importance of multidimensional frailty. The main objective of this study is to evaluate in hospitalised older persons with AF the benefit/risk ratio of the anticoagulant treatments, considering the severity of frailty, determined by the multidimensional prognostic index (MPI).

**Methods:**

In this European, multicentre, prospective study, older hospitalised patients (≥65 years) with non-valvular AF were followed-up for 12 months. Anticoagulants’ use at discharge ascertained using medical records. MPI was calculated using tools derived from comprehensive geriatric assessment, classifying participants in robust, pre-frail or frail. Mortality (primary outcome); vascular events, including ischemic heart disease or ischemic stroke, hemorrhagic stroke or gastrointestinal bleedings (secondary outcomes).

**Results:**

2,022 participants (mean age 82.9 years; females 56.6%) were included. Compared with people not taking anticoagulants (*n* = 823), people using vitamin K antagonists (*n* = 450) showed a decreased risk of mortality (hazard ratio, HR = 0.74; 95% CI: 0.59–0.93), more pronounced in patients using direct oral anticoagulants (DOACs) (*n* = 749) (HR = 0.46; 95% CI: 0.37–0.57). Only people taking DOACs reported a significantly lower risk of vascular events (HR = 0.55; 95% CI: 0.31–0.97). The efficacy of DOACs was present independently from frailty status. The risk of gastrointestinal bleedings and hemorrhagic stroke did not differ based on the anticoagulant treatments and by MPI values.

**Conclusions:**

Anticoagulant treatment, particularly with DOACs, was associated with reduced mortality in older people, without increasing the risk of hemorrhagic events, overall suggesting the importance of treating with anticoagulants older people with AF.

## Key Points

- Atrial fibrillation is a common condition in older people, but the role of frailty is still unknown.
- In our study, anticoagulant treatment, particularly with direct oral anticoagulants (DOACs), was associated with reduced mortality in older people.
- Future studies are needed to integrate the multidimensional evaluation in the management of atrial fibrillation.

## Introduction

The prevalence of atrial fibrillation (AF) linearly increases with age, being associated with several unfavourable outcomes [1]. Several randomised clinical trials (RCTs) demonstrated that anticoagulant treatment was effective in preventing ischemic stroke and reducing mortality rates in older patients with AF [1]. Nevertheless, the translation of these guidelines into clinical practice remains a challenge in geriatric medicine [2]. In fact, the rate of anticoagulant prescribing in older participants with AF is less than 50%, despite a clear indication [3].

The results of several RCTs indicate that DOACs (Direct Oral Anticoagulants) are at least as effective and safe as vitamin K antagonists (VKAs) but offer significant simplification of the therapy for stroke prevention in AF [4]. Most of clinicians, however, have questioned the generalizability of these results to older people at highest risk, i.e. frail and multimorbid older participants, since these individuals were not represented in large RCTs [5]. A recent study, among about 1,000 very old subjects affected by AF, reported that a low dose of edoxaban was superior to placebo in preventing stroke or systemic embolism and did not result in a significantly higher incidence of major bleeding than placebo [6]. However, the importance of prognosis was not incorporated in this study. Thus, ongoing studies are needed to further inform on the efficacy and safety of anticoagulants in a ‘real-world setting’, especially in patients with poor prognosis [7].

To better evaluate the benefit and burdens of treatments in the frail older participants, many guidelines recommend incorporating clinical decision-making tools based on a comprehensive geriatric assessment (CGA) for taking decisions in clinical practice [8]. The Multidimensional Prognostic Index (MPI) is a widely used prognostic index for estimating both short- and long-term mortality, easily derived on information gathered from a CGA [9–14]. Initially developed and validated in hospitalised older patients [9], a series of multicentre studies, actually involving more than 60,000 older participants across different settings and medical conditions, reported that the MPI is an accurate and well calibrated tool for predicting mortality and other negative health outcomes [9–13, 15]. MPI shows a high validity, reliability and feasibility for the management of older persons with different degrees of complexity [16]. Regarding anticoagulant therapy, a retrospective observational data of 1,827 older community-dwellers with AF showed that patients with higher mortality risk, as evaluated by the MPI, were less treated with anticoagulants than patients with lower mortality risk, even if frailer patients had a similar benefit in term of mortality reduction from the anticoagulant therapy [17].

The main objective of the EUROSAF (EURopean study of Older Subjects with Atrial Fibrillation) study is to prospectively evaluate in a population of hospitalised older participants with non-valvular AF the clinical benefit/risk ratio of the anticoagulant treatments in terms of mortality, thromboembolic events and bleeding side-effects over 1 year of follow-up. Moreover, we aimed to evaluate whether a different prognostic profile, as determined by the MPI, is associated with differences in mortality, thromboembolic events and side effects including bleeding events.

## Materials and methods

The study protocol [18] was previously registered in ClinicalTrials.gov (https://clinicaltrials.gov/ct2/show/NCT02973984). Other details are reported in https://www.eurosaf.eu/home.html.

### Study population and inclusion criteria

EUROSAF is an international, multicentre, prospective, observational study involving older participants (defined as those aged ≥65 years) affected by non-valvular AF hospitalised in 24 different European geriatric centres from 12 European countries (Austria, Belgium, Czech Republic, Finland, France, Germany, Italy, Poland, Portugal, Slovakia, Spain, The Netherlands). The study was an activity of the Special Interest Group on CGA of the EuGMS (European Geriatric Medicine Society) [18]. The diagnosis of AF was made using ECG recordings integrated with medical records available for each centre.

All consecutive patients admitted to the Geriatrics Units involved in the study were evaluated. The inclusion criteria were patients of both genders, aged >65 years, admitted to hospital for any reason, a documented diagnosis of non-valvular AF, able to give their informed consent. Patients not able to provide informed consent or deceased during hospitalisation were excluded. The enrollment period lasted from 01 January 2016 to 31 December 2020.

Ethical approval: The ethical committees of each centre formally approved this study. The ethical committee of the leading centre (Ente Ospedaliero Genova) formally approved the study on 08 June 2016, protocol 162REG2016. The other ethical committees approved, for each centre, the study. Written informed consent was given by participants who underwent initial evaluation and/or their proxies for their clinical records to be used in this study. All patient records and information were anonymised and de-identified prior to the analysis.

### Anticoagulants’ prescription

Participants were divided in three categories according to the prescription of anticoagulants at the discharge. Vitamin K antagonists (VKAs) included warfarin, acenocoumarol, dicoumarol and phenindione, while DOACs included dabigatran, rivaroxaban, apixaban and edoxaban, according to the ATC code. Participants not taking VKAs or DOACs were categorised as no anticoagulant treatment.

### The multidimensional prognostic index

In order to develop an MPI that correctly reflects the multidimensional impairment of a hospitalised geriatric patient, a cluster analysis on CGA data of the development cohort population was initially made for evaluating the independence of several factors commonly used in CGA in predicting mortality [9]. At hospital discharge, the MPI derived from information obtained through a standard CGA that considered these domains [9]

- Activities of daily living (ADL) index, which defines the level of dependence/independence in six daily personal care activities (bathing, toileting, feeding, dressing, urine and bowel continence and transferring (in and out of bed or chair));
- Instrumental Activities of Daily Living (IADL) considering eight activities that are more cognitively and physically demanding than ADL, i.e. managing finances, using telephone, taking medications, shopping, using transportation, preparing meals, doing housework and washing;
- Short Portable Mental Status Questionnaire (SPMSQ), a 10 item questionnaire investigating orientation, memory, attention, calculation and language; validated versions were used in each local language.
- Cumulative Illness Rating Scale (CIRS) that uses a 5-point ordinal scale (score 1–5) to estimate the severity of pathology in each of 13 systems, including cardiac, vascular, respiratory, eye–ear–nose–throat, upper and lower gastrointestinal, hepatic, renal, genitourinary, musculoskeletal, skin disorder, nervous system, endocrine-metabolic and psychiatric behavioral disorders. Based on the ratings, the Comorbidity Index (CIRS-CI) score, which reflects the number of concomitant diseases, was derived from the total number of categories in which moderate or severe levels (grade from 3 to 5) of disease were identified (range from 0 to 13).
- Mini Nutritional Assessment (MNA) short form (SF), a brief questionnaire comprising anthropometric measurements combined with a questionnaire regarding loss of appetite, recent weight loss, mobility, acute distress and neuropsychological problems.
- Exton Smith Scale (ESS), a five items questionnaire determining physical and mental condition, activity, mobility and incontinence indicating the risk of pressure sores.
- Number of medications taken at the hospital discharge.
- Cohabitation status divided as living alone, in an institution, or with family members.

For each domain, a tripartite hierarchy was used, i.e. 0 = no problems, 0.5 = minor problems and 1 = major problems, based on conventional cut-off points derived from the literature for each item [15]. The sum of the calculated scores from the eight domains was divided by eight to obtain a final MPI risk score ranging from 0 = no risk to 1 = higher risk of mortality [15]. Traditionally, the division of MPI is made using three categories, i.e. MPI-1 (low risk of mortality, robustness) <0.33; MPI-2 (intermediate risk, pre-frailty) between 0.33 and 0.66 and MPI-3 (high risk, frailty) with an MPI value >0.66. The execution of MPI requires, in mean, 15 min [19]. At the following address: https://multiplat-age.it/index.php/en/tools, it is possible to download for free the software. In Supplementary Table 1, available in *Age and Ageing* online, we reported how MPI is built.

### Clinical evaluations

Information regarding the systemic thromboembolic risk by using the CHA2DS2-Vasc score (congestive heart failure, hypertension, age category, diabetes, stroke, vascular disease, gender) and the bleeding risk by using the HAS-BLED score (hypertension, abnormal liver or renal function, stroke, bleeding, labile INR, old age, drugs or alcohol) were also collected. Main and secondary diagnoses at discharge were coded using the ICD 10, as well as all prescribed medications at hospital discharge categorised using the ATC code.

### Follow-up evaluations

During the follow-up period, at 6 and 12 months, the following information was collected based on hospital re-admissions and death certificates. Mortality status, with the date and cause of death, categorised using the ICD 10 code, was considered the primary outcome. Secondary outcomes were defined using either medical records or death certificates

- Vascular events, including ischemic heart disease (ICD 10 code I20-I25) or ischemic stroke (I63-I65);
- Hemorrhagic stroke (I61);
- Gastrointestinal bleedings (K92).

### Statistical analysis

The different variables considered were evaluated both overall and for single centre and the presence of heterogeneity across centres was checked without identifying this problem. The demographic and clinical characteristics of the patients were reported as mean and standard deviation or frequency and percentage for continuous and categorical variables, respectively. Between-group comparisons were performed using the T-test independent samples for continuous variables and the Pearson chi-square test for categorical ones, by survival status. The normality of distribution of continuous variables was investigated by using the Kolmogorov–Smirnov test.

Endpoints count was analysed using the Poisson model within a generalised mixed-effects model for better accounting the possible heterogeneity across centres and using as offset the effective period of follow-up and reporting the data as incidence rates along their 95% confidence intervals (95% CI). Time to event was calculated as time between the hospital discharge and primary or secondary outcomes of interest, whichever came first.

**Table 1:** Baseline characteristics by survival status

Parameter	Alive (*n* = 1,509)	Dead (*n* = 513)	*P*-value
Age (mean, SD)	82.2 (7.4)	85.1 (7.7)	<0.0001
Females (%)	58.2	52.2	0.02
No anticoagulants (%)	36.0	54.6	<0.0001
Vitamin K antagonists (%)	22.6	21.2	0.51
Direct-Acting Oral Anticoagulants (%)	41.4	24.2	<0.0001
CHA2DS2-VASC (mean, SD)	4.8 (1.5)	5.0 (1.5)	0.10
HAS-BLED (mean, SD)	2.7 (1.1)	2.9 (1.2)	<0.0001
Short portable mental state questionnaire (mean, SD)	2.5 (2.7)	3.9 (3.3)	<0.0001
Exton-Smith Scale (mean, SD)	16.3 (3.0)	14.1 (3.7)	<0.0001
Activities of daily living (mean, SD)	4.0 (2.1)	2.8 (2.3)	<0.0001
Instrumental activities of daily living (mean, SD)	4.0 (2.8)	2.8 (2.3)	0.001
Cumulative Illness Rating Scale-Comorbidity Index (mean, SD)	3.9 (2.2)	4.6 (2.3)	<0.0001
Mini Nutritional Assessment-Short Form (mean, SD)	9.8 (2.9)	8.1 (3.1)	<0.0001
Number of drugs (mean, SD)	7.6 (3.3)	8.0 (3.2)	0.02
Alone (%)	29.4	23.8	<0.0001
MPI (mean, SD)	0.45 (0.20)	0.58 (0.20)	<0.0001

Abbreviations: CHA2DS2-VASC: congestive heart failure, hypertension, age category, diabetes, stroke, vascular disease, sex category; HAS-BLED. hypertension, abnormal liver or renal function, stroke, bleeding, labile INR, old age, drugs or alcohol.

The association between anticoagulants’ treatment at discharge and the outcomes of interest was analysed using number needed to treat (NNT) and number needed to harm (NNH). After verifying the assumptions needed for this analysis, a Cox’s regression analysis, adjusting for age, sex, centre, MPI, CHA2DS2-VASC score and HAS-BLED score. In the case of mortality as outcome, data were censored to the last observation available for alive patients or if the patients not initially taking anticoagulants took during the follow-up period. Patients not taking anticoagulants were taken as reference group. Moreover, to test the importance of multidimensional evaluation in the association between anticoagulants and outcomes of interest, we stratified our analyses by MPI categories. For secondary outcomes, patients dead for reasons other than the secondary outcome examined, were censored. The data were reported as hazard ratio (HR) along their 95% CIs.

A *P*-value <0.05 was considered statistically significant. All statistical analyses were performed using SPSS software (version 26.0) and with MedCalc (version 22.09), considering the Bonferroni’s correction, i.e. putting the threshold to 0.017.

## Results

Among 2,166 initially enrolled, 58 participants were excluded since MPI was not calculable and 86 died during the first hospitalisation, finally leaving 2,022 patients eligible for this study.

**Table 2:** Association between anticoagulation therapy and primary and secondary outcomes of the EUROSAF study

	Number of events/participants	Number needed to treat or number needed to harm	Incidence rate, per 100,000 persons-years	HR^a^ (95% CI)	*P*-value
**Mortality (*n* = 513, incidence rate 85 per 100,000 persons-years)**					
No anticoagulation	280/823	–	127 (113–143)	1, reference	–
VKAs	109/450	10	79 (65–95)	0.74 (0.59–0.93)	0.009
DOACs	124/749	6	51 (42–61)	0.46 (0.37–0.57)	<0.0001
**Vascular events (*n* = 62, incidence rate 10 per 100,000 persons-years)**					
No anticoagulation	30/823	–	14 (10–20)	1, reference	–
VKAs	11/450	19	8 (4–15)	0.54 (0.27–1.08)	0.08
DOACs	21/749	20	8 (6–13)	0.55 (0.31–0.97)	0.04
**Gastrointestinal bleedings (*n* = 54, incidence rate 9 per 100,000 persons-years)**					
No anticoagulation	22/823	–	10 (7–15)	1, reference	–
VKAs	11/450	19	8 (4–15)	0.91 (0.44–1.91)	0.81
DOACs	21/749	20	8 (6–13)	0.95 (0.51–1.75)	0.86
**Hemorrhagic stroke (*n* = 19, incidence rate 3 per 100,000 persons-years)**					
No anticoagulation	6/823	–	3 (1–6)	1, reference	–
VKAs	6/450	167	4 (2–9)	1.50 (0.48–4.97)	0.49
DOACs	7/749	435	3 (1–6)	1.04 (0.34–3.17)	0.95

^a^HRs are reported with their 95% CIs and corresponding *P*-values, after adjusting for age, sex, centre, MPI, CHA2DS2-VASC (congestive heart failure, hypertension, age category, diabetes, stroke, vascular disease, sex category) score, HAS-BLED (hypertension, abnormal liver or renal function, stroke, bleeding, labile INR, old age, drugs or alcohol) score.

**Figure 1:**
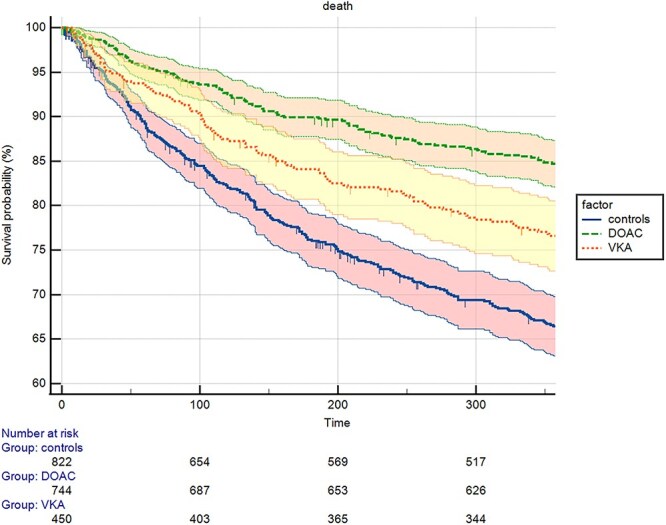
Association between anticoagulation status at discharge and mortality, over 1 year of follow-up. Survival curves are reported with the correspondent 95% CIs

The 2,022 patients aged a mean of 82.9 ± 7.6 years (range: 65–104) and were, mainly females 56.6%, affected by AF. Table 1 shows the baseline characteristics of the participants by survival status. Using a Student *T*-test for independent samples or a chi-square test, compared with the 1,509 participants alive, the 513 patients who dead during the follow-up were significantly older and more frequently males. Participants who dead during follow-up reported a significantly lower proportion of DOACs (24.2 versus 41.4, *P* < 0.0001), but a similar rate of VKAs (*P* = 0.51). People who dead did not differ in terms of CHA2DS2-VASC score compared with their counterparts but reported significantly higher scores in HAS-BLED. Finally, when considering multidimensional frailty domains, participants who died reported a significant higher impairment in all domains considered (*P* < 0.05), finally leading to a higher MPI score (0.58 ± 0.20 versus 0.45 ± 0.20; *P* < 0.0001) (Table 1).

Over 1 year of follow-up, 513 deaths (incidence rate 85 per 100,000 persons-years), 62 vascular events (incidence rate 10 per 100,000 persons-years), 54 gastrointestinal bleedings (incidence rate 9 per 100,000 persons-years) and 19 hemorrhagic strokes (incidence rate 3 per 100,000 persons-years) were observed (Poisson’s model) (Table 2).

Compared with no anticoagulation, patients taking VKAs showed a decreased risk of mortality (HR = 0.74; 95% CI: 0.59–0.93; *P* = 0.009) that was more pronounced in patients using DOACs (HR = 0.46; 95% CI: 0.37–0.57; *P* < 0.0001) (Figure 1, Table 2). On average, the NNT with DOACs to prevent mortality in 1 patient was 6; for VKAs, we should treat 10 patients for preventing mortality in one older patient with AF (Table 2). When analysing for single medications, practically all anticoagulants led to a decreased risk of death (Supplementary Table 2 available in *Age and Ageing* online). However, only patients taking DOACs reported a lower risk of vascular events (HR = 0.55; 95% CI: 0.31–0.97; *P* = 0.04, Supplementary Figure 1), even if not statistically significant considering the Bonferroni’s correction, while the use of VKAs was not (*P* = 0.08). Finally, no significant differences across the three treatment groups were observed in terms of gastrointestinal bleedings (Supplementary Figure 2) or hemorrhagic stroke (Supplementary Figure 3) (Table 2).

Table 3 shows the association between anticoagulant therapy and outcomes of interest, stratified by the grade of multidimensional frailty as assessed by the MPI. Overall, the efficacy of DOACs was independent from the grade of multidimensional frailty, even if we observed a trend in the NNT that was 9 in MPI 1 group, 7 in MPI 2 group and 4 in MPI 3 group. The use of DOACs was associated with a decreased risk of vascular events only in robust patients (MPI 1 group) (HR = 0.18; 95% CI: 0.05–0.67; *P* = 0.0002) (NNT = 29), while no significant effect was observed in frailer patients. The risk of gastrointestinal bleedings and hemorrhagic stroke did not differ based on the anticoagulant treatments and by MPI values (Table 3).

**Table 3:** Association between anticoagulation therapy and primary and secondary outcomes of the EUROSAF study by MPI values.

	MPI 1 (*n* = 570)			MPI 2 (*n* = 951)			MPI 3 (*n* = 502)		
Outcome	No treatment	VKAs	DOACs	No treatment	VKAs	DOACs	No treatment	VKAs	DOACs
*Number needed to treat for mortality*	–	*23*	*9*	–	*26*	*7*	–	*6*	*4*
Mortality	1 [ref.]	0.72 (0.42–1.24) *P* = 0.24	0.42 (0.23–0.74) *P* = 0.003	1 [ref.]	0.78 (0.55–1.09) *P* = 0.78	0.42 (0.30–0.60) *P* < 0.0001	1 [ref.]	0.67 (0.45–1.00) *P* = 0.047	0.50 (0.36–0.70) *P* < 0.0001
* Number needed to treat for vascular events*	–	*30*	*29*	–	*200*	*63*	–	*250*	*28*
Vascular events	1 [ref.]	0.19 (0.04–0.86) *P* = 0.03	0.18 (0.05–0.67) *P* = 0.0002	1 [ref.]	0.78 (0.31–1.98) *P* = 0.61	0.58 (0.23–1.43) *P* = 0.24	1 [ref.]	0.38 (0.05–3.09) *P* = 0.36	1.26 (0.45–3.50) *P* = 0.66
* Number needed to harm for gastrointestinal bleeding*	–	*91*	*333*	–*-*	*143*	*1,000*	–	*500*	*56*
Gastrointestinal bleedings	1 [ref.]	1.37 (0.30–6.28) *P* = 0.69	0.78 (0.15–4.01) *P* = 0.76	1 [ref.]	0.70 (0.21–2.28) *P* = 0.55	0.85 (0.33–2.19) *P* = 0.73	1 [ref.]	0.73 (0.20–2.74) *P* = 0.64	0.99 (0.38–2.56) *P* = 0.99
* Number needed to harm for hemorrhagic stroke*	–	–	–	–	–	–	–	–	–
Hemorrhagic stroke	1 [ref.]	Not possible	Not possible	1 [ref.]	2.11 (0.50–9.00) *P* = 0.31	1.30 (0.30–5.66) *P* = 0.72	1 [ref.]	Not possible	0.38 (0.04–3.87) *P* = 0.38

^a^HRs are reported with their 95% CIs and corresponding *P*-values, after adjusting for age, sex, centre, CHA2DS2-VASC (congestive heart failure, hypertension, age category, diabetes, stroke, vascular disease, sex category) score, HAS-BLED (hypertension, abnormal liver or renal function, stroke, bleeding, labile INR, old age, drugs or alcohol) score.

## Discussion

In the EUROSAF study, we found that anticoagulant treatment, particularly DOACs, was associated with reduced mortality independently from their frailty status, without a significant increase in incident hemorrhagic events.

A first important finding of our work is that the anticoagulant treatment, particularly DOACs, is associated with a reduction in mortality, independently from the presence of multidimensional frailty assessed by the MPI. A large retrospective study made among Medicare beneficiaries in the United States reported that among older adults with AF, compared with VKAs, DOACs were associated with a reduced risk of death, ischemic stroke or major bleeding, particularly in robust participants [20]. Our study partially confirmed these findings since the use of DOACs was associated with a decreased risk of death independently from the presence and severity of multidimensional frailty, while a reduced risk of vascular events was observed only in robust patients. Other studies confirmed the beneficial effect of anticoagulants in older patients, also in frailer patients affected by AF [21, 22]. Even if these studies advanced our knowledge regarding this topic, we believe that EUROSAF study adds some important concepts including the prospective study design that permits to decrease the selection bias. From a clinical perspective, we believe that a multidimensional representation of frailty, according to its impaired domains, permits to clinicians to highlight domains needing specific interventions, also in older people affected by AF [23]. For example, a patient with impairments in nutritional domain can have benefit from a consultation with a dietician. Similarly, in people affected by AF, we can hypothesise that CGA clinics and cardiac rehabilitation programmes can improve patient outcomes, in particular functional capacity [23].

Moreover, in our study, about half of the patients did not take any anticoagulant therapy at hospital discharge. Of importance, people with higher MPI scores, indicating a higher presence of multidimensional frailty, were less frequently treated with anticoagulants confirming previous reports of a sub-optimal prescription of oral anticoagulants in frail older patients with AF [3]. The rate of older people for which anticoagulant therapy is not prescribed remains high, despite the evidence of a beneficial effect in these patients [24].

Compared with patients not taking anticoagulation treatment, older patients taking DOACs reported a reduction in the risk of all-cause mortality of over 50%, while patients taking VKAs had a significant reduced risk in overall death of about 26%. First, we can argue that DOACs are more efficacious because of more robust anticoagulation than with VKAs [25]. Moreover, it is possible that anticoagulant therapy, particularly DOACs, had some pleiotropic effects. Recent literature reported that DOACs can have an anti-atherosclerotic effect [26]. Moreover, it seems that DOACs may contribute to the prevention of cardiac remodelling by reducing the processes of inflammation and fibrosis [27]. Finally, it was postulated that FXa inhibitors are shown to increase the expression of vascular growth factors, stimulate the migration of endothelial progenitor cells and improve their function, thus manifesting their angiogenic effect [28].

Another important finding of our study is that anticoagulant treatment was not associated with a significant higher risk of bleedings that are among the most important factors in not prescribing anticoagulant treatment in older people affected by AF [29]. Contrary to previous observations [30], in the EUROSAF study that includes older patients having a high rate of multimorbidity, polypharmacy and other common geriatric syndromes, the use of anticoagulants was not associated with a higher risk of bleedings leading to mortality or hospitalisations. Therefore, our findings, based on a prospective study specifically designed for reaching these outcomes, further supported that not treating older people only based on a hypothetical bleeding risk is probably not longer justified.

The findings of our study must be interpreted within its limitations. First, the patients included were hospitalised: it is therefore possible that the inclusion of patients in different settings may lead to different findings. Decisions on drug treatments for chronic conditions in a setting of acute disease may be different. Second, the standard CGA was calculated only at discharge: people died during hospital stay that could be frailer, possibly introducing a selection bias. Third, the observational nature of the study that, however, can better address the important problem of including frailer people in this literature. In the absence of randomisation, in fact, some situational factors and the preferences of clinicians may have influenced the choice of anticoagulants. Finally, when stratified by MPI classes, we observed a low power for some secondary outcomes, particularly for participants taking VKAs. At the same time, the incidence of the secondary outcomes of the EUROSAF study is similar, when standardised, to other epidemiological studies made in older people. [1]

In conclusion, the EUROSAF study reports that anticoagulant treatment, particularly with DOACs, was associated with reduced mortality in older people without a significant increase in incident hemorrhagic events, across different grades of multidimensional frailty. Our findings suggest that more older people should benefit from the use of anticoagulants and that physicians should not be reluctant to use them in very old and complex patients. Future intervention studies are needed to confirm our findings.

## Supplementary Material

aa-23-0470-file003_afad216

## Data Availability

The data are available upon reasonable request to the Corresponding Author.

## The EUROSAF Study Investigators' List Include

Alberto Ferri, Alessandra Argusti, Federica Gandolfo, Clarissa Musacchio, Katerin Leslie Quispe Guerrero, Alberto Pilotto, Carlo Custodero, Vincenzo Solfrizzi, Carlo Sabbà, Maria Cristina Polidori, Joshua Verleysdonk, Nico Noetzela, Timo Strandberg, Juhani Rossinen, Laura Pikkarainen, Tuomo Nieminen, Eva Topinkova, Helena Michalkova, Pavla Madlova, Lucie Bautzka, Stefania, Nicola Ferrara, Lucia Gioia, Anna Maria Iannicelli, Mario Barbagallo, Nicola Veronese, Giovanna Di Bella, Federica Cacioppo, Giovanni Ruotolo, Alberto Castagna, Regina Roller-Wirnsberger, Christian Sebesta, Sonja Lindner, Alfonso Cruz-Jentoft, Luisa A. Hernandez-Sanchez, Jana Albeniz Lopez, Genesis Estefanıa Olaya-Loor, Pedro Marques da Silva, Heidi Gruner, Jean Petermans (deceased), Sophie Gillain, Veronique Jonart, Ondrej Vyska, Jiri Nakladal, Katarina Bielakova, Hana Matejovska-Kubesova, Adrian Enica, Stephanie Roth, Benjamin Jacquet, Vito Curiale, Nicolas Berg, Livia Mirea Cimpeanu, Rafaela Verissimo, Leonor Silva, Luciana Silva, Pedro Magalhães, Gabriel Ioan Prada, Anna Marie Herghelegiu, Catalina Raluca Nuta, Blanca Garmendia-Prieto, Isabel Lozano-Montoya, Javier Jaramillo-Hidalgo, Javier Gomez-Pav, Ursula Müller-Werdan, Gordon Werth, Adrian Rosada, Ozlem Yilmaz, Sena Geurkaş Francesco Mattace-Raso. All these researchers gave the consensus to be cited in the collaborative group of the EUROSAF study.
