# Supplementary material for: Frailty and anticoagulants in older subjects with atrial fibrillation: the EUROSAF study
Source: Age Ageing. 2023 Nov 27;52(11):afad216. doi: 10.1093/ageing/afad216 (PMC11373113; doi:10.1093/ageing/afad216)
Supplement: aa-23-0470-file003_afad216 [file aa-23-0470-file003_afad216.docx]

**Supplementary Table 1. Multidimensional prognostic index**

The Multidimensional Prognostic Index (MPI) is a prognostic tool based on a standardized CGA which allows to predict short- and long-term mortality in elderly subjects.

MPI includes eight domains as follows:

1) Activities of daily life (ADL - Activities of Daily Living): composed of 6 items to evaluate the patient's functional status in daily life activities.

2) Instrumental activities of daily living (IADL - Instrumental Activities of Daily Living): composed of 8 items that evaluate the level of independence in the instrumental activities of daily life.

3) Short Portable Mental Status Questionnaire (SPMSQ): composed of 10 questions which assessed the patient's cognitive status, in particular spatio-temporal and personal orientation, memory, attention and computation ability.

4) Exton-Smith Scale (ESS): composed of 5 items for assessing the risk of developing pressure sores.

5) Nutritional Assessment (MNA, Mini-Nutritional Assessment) -short form: it consists of 6 items to assess the patient's nutritional status through three types of assessments: anthropometric assessment, dietary assessment, and subjective assessment.

6) Cumulative Illness Rating Scale (CIRS): consisting of 14 items to describe the pathologies present at the time of patient recruitment, identifying their severity by calculating the comorbidity and severity index respectively.

7) Number of drugs in use: a single item that allows you to quantify the number of drugs taken by the patient upon entry in the ward.

8) Cohabitation status: it identifies the patient's socio-housing condition.

To each domain is assigned a score corresponding to a risk, respectively:

- Low (value of 0);

- Moderate (value of 0.5);

- High (value of 1).

The following table shows the ranges of values ​​for each domain corresponding to the three risk levels.

| **MPI - Multidimensional Prognostic Index** | | | |
| --- | --- | --- | --- |
|  | **Score in each domain** | | |
|  | **Low risk**  **(Value = 0)** | **Moderate risk**  **(Value = 0.5)** | **High risk**  **(Value = 1)** |
| 1. **ADL** | 6-5 | 4-3 | 2-0 |
| 1. **IADL** | 8-6 | 5-4 | 3-0 |
| 1. **SPMSQ** | 0-3 | 4-7 | 8-10 |
| 1. **ESS** | 16-20 | 10-15 | 5-9 |
| 1. **MNA-SF** | 12-14 | 8-11 | 0-7 |
| 1. **CIRS** | 0 | 1-2 | ≥3 |
| 1. **Number of drugs** | 0-3 | 4-6 | ≥7 |
| 1. **Cohabitation status** | Family | Nursing home | Alone |
| Add the values of the single items and divide by 8 | | **TOTAL SCORE MPI** | 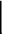 |

The MPI is expressed as a continuous value from 0.0 (lowest risk of mortality) to 1.0 (highest risk of mortality). Appropriate validated cut-offs have been calculated to identify three levels of mortality risk as follows:

1. MPI-1: mild risk (MPI values from 0.0 to 0.33),
2. MPI-2: moderate risk (MPI values from 0.34 to 0.66),
3. MPI-3: severe risk (MPI values from 0.67 to 1.0).

The calculation of the final MPI will be made through the software application available for free at the https://multiplat-age.it/index.php/en/tools.

Supplementary Table 2. Effect of single anticoagulant medication on mortality

| Overall mortality |  | HR* | 95.0% CI per Exp(B) | |  |
| --- | --- | --- | --- | --- | --- |
|  | Sample size |  | Inferiore | Superiore | p-value |
| No anticoagulant treatment | 824 | 1 | 1 | 1 | reference |
| Warfarin | 315 | 0.753 | 0.572 | 0.990 | 0.042 |
| Acenocumarol | 136 | 0.715 | 0.504 | 1.014 | 0.060 |
| Dabigatran | 107 | 0.572 | 0.358 | 0.912 | 0.019 |
| Rivaroxaban | 213 | 0.497 | 0.346 | 0.714 | 0.000 |
| Edoxaban | 53 | 0.415 | 0.205 | 0.839 | 0.014 |
| Apixaban | 375 | 0.419 | 0.316 | 0.557 | <0.0001 |

***** Hazard ratios (HRs) are reported with their 95% confidence intervals (CIs) and corresponding p-values, after adjusting for age, sex, center, multidimensional prognostic index, CHA2DS2-VASC (congestive heart failure, hypertension, age category, diabetes, stroke, vascular disease, sex category) score, HAS-BLED (hypertension, abnormal liver or renal function, stroke, bleeding, labile INR, old age, drugs or alcohol) score

Supplementary Figure 1. Association between anticoagulation status at discharge and vascular events, over one year of follow-up.


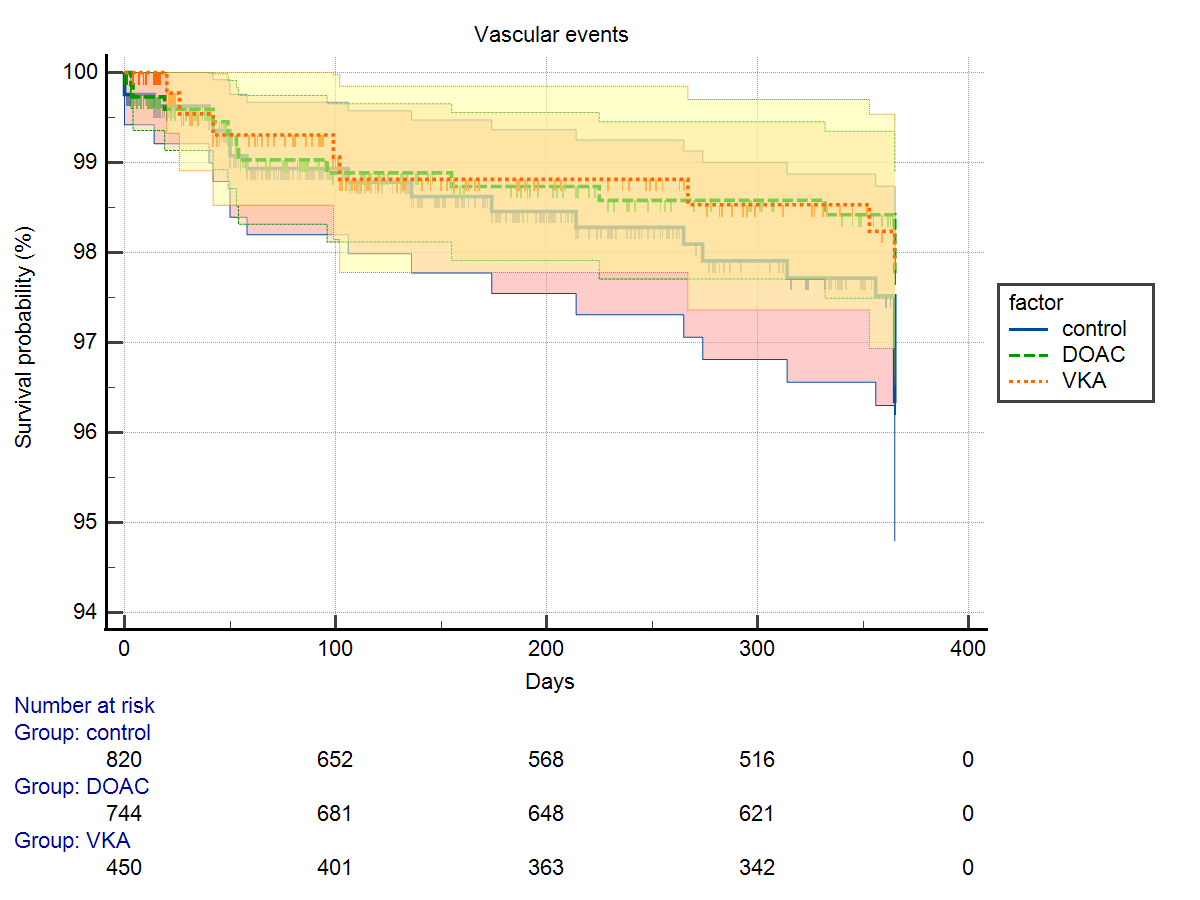


Survival curves are reported with the correspondent 95% confidence intervals

Supplementary Figure 2. Association between anticoagulation status at discharge and gastrointestinal bleedings, over one year of follow-up.


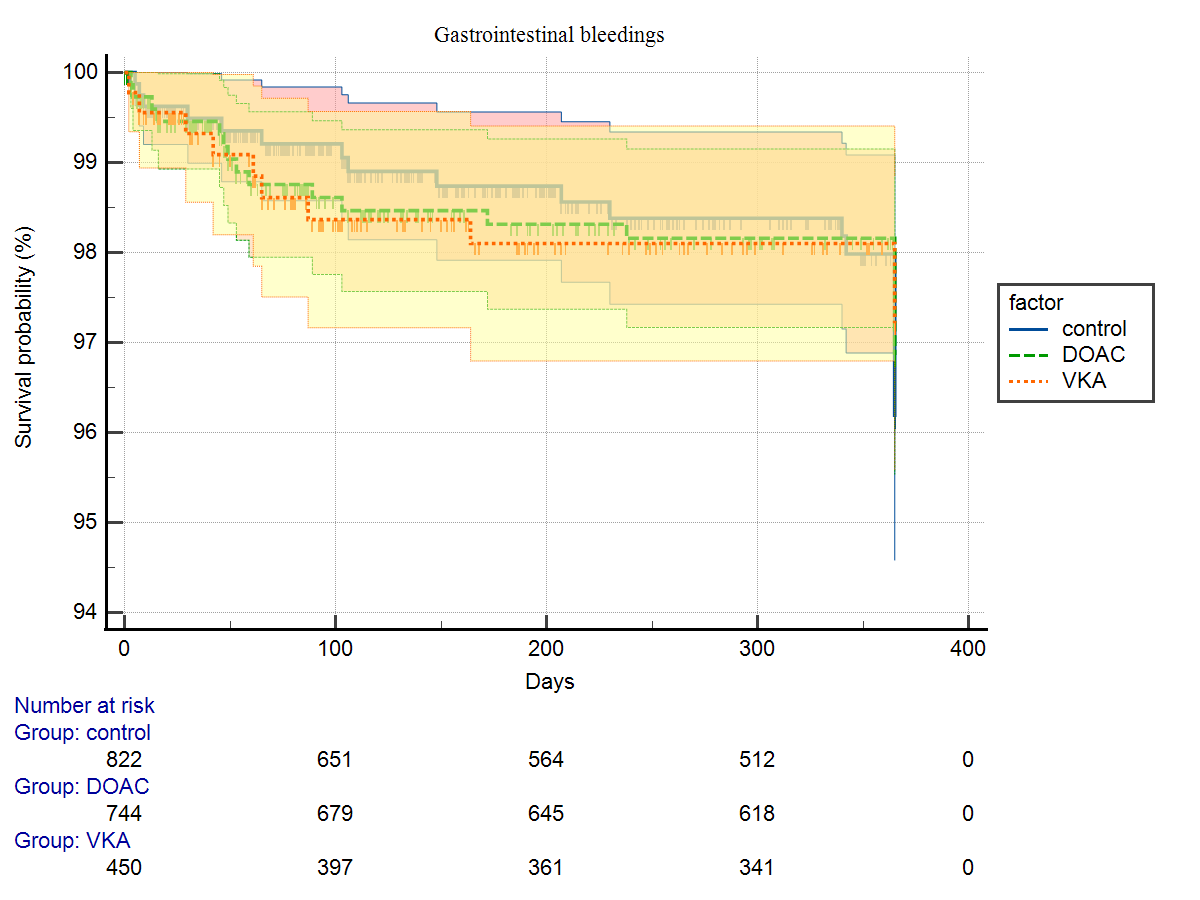


Survival curves are reported with the correspondent 95% confidence intervals

Supplementary Figure 3. Association between anticoagulation status at discharge and hemorrhagic stroke, over one year of follow-up.


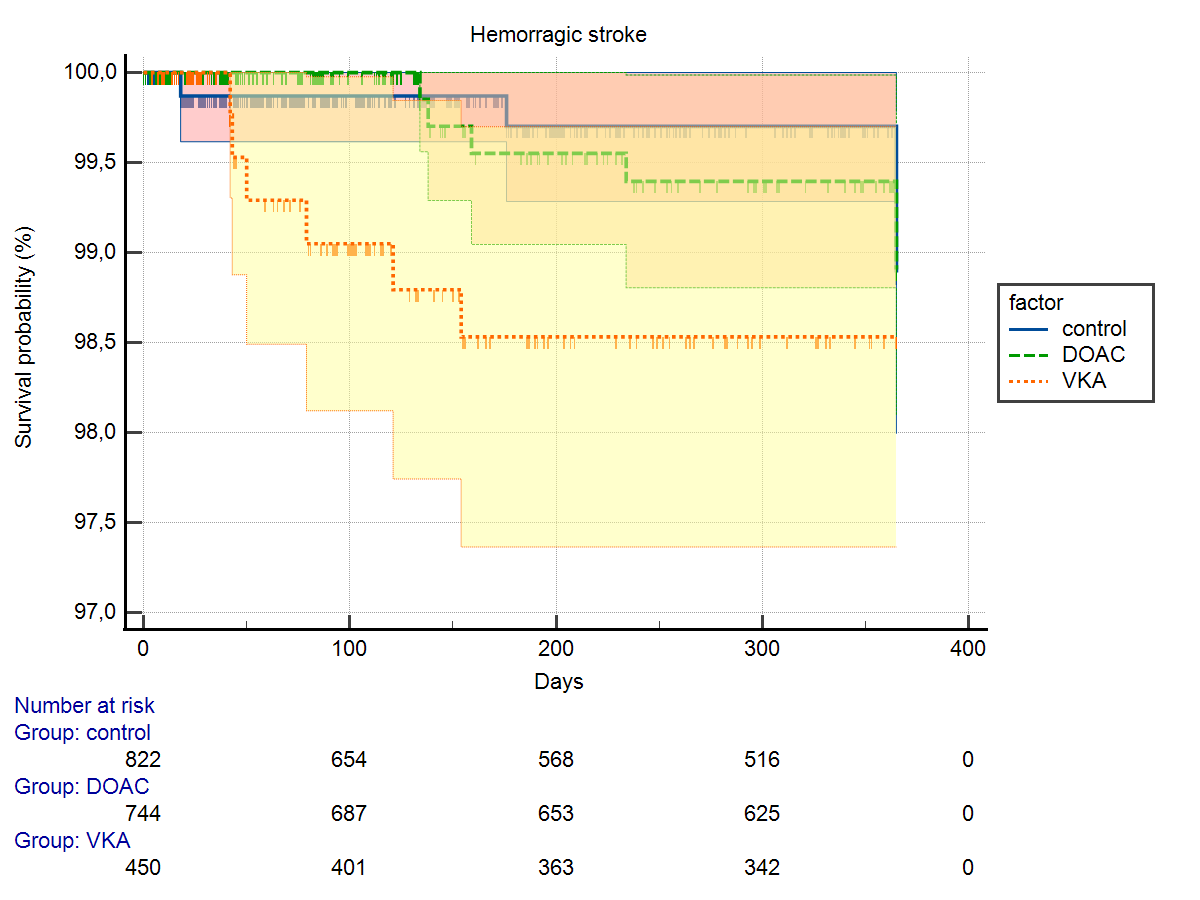


Survival curves are reported with the correspondent 95% confidence intervals
